# Lack of Variation at Phosphoglucose Isomerase *(Pgi)* in Bumblebees: Implications for Conservation Genetics Studies

**DOI:** 10.1371/journal.pone.0065600

**Published:** 2013-06-04

**Authors:** Jonathan S. Ellis, Lucy M. Turner, Mairi E. Knight

**Affiliations:** 1 School of Science and the Environment, Manchester Metropolitan University, Manchester, Greater Manchester, United Kingdom; 2 School of Marine Science and Engineering, University of Plymouth, Plymouth, Devon, United Kingdom; 3 School of Biomedical and Biological Sciences, University of Plymouth, Plymouth, Devon, United Kingdom; Oxford Brookes University, United Kingdom

## Abstract

Assessing genetic variation underlying ecologically important traits is increasingly of interest and importance in population and conservation genetics. For some groups generally useful markers exist for examining the relative role of selection and drift in shaping genetic diversity e.g. the major histocompatibility complex in vertebrates and self-incompatibility loci in plants. For invertebrates there is no such generally useful locus. However, phosphoglucose isomerase (*Pgi*) has been proposed as a useful functional marker in the conservation genetics of invertebrates. Where thermal microclimate varies, balanced polymorphisms may be maintained due to trade-offs between thermally stable and kinetically advantageous allelic forms. We here report very low levels of *Pgi* variation in bumblebees rendering this locus to be of little use as an adaptive marker in a conservation genetics context in this group. Potential explanations for this lack of variation are considered.

## Introduction

Over recent years conservation genetics has become a fully established empirical discipline [1]. Having largely focussed on assessing stochastic and demographic processes using neutral markers, lately there has been growing interest in assessing functional adaptive genetic variation underlying traits of ecological importance [1], [2], [3] as potentially more relevant measures of genetic viability, particularly in some conservation contexts [e.g. 4].

A goal of modern conservation genetics is to address the extent to which adaptive variation, especially for loci where polymorphism should be maintained, is affected by the forces known to affect neutral diversity, in small and/or isolated populations [1]. Assessing the relative roles of selection and drift on genetic diversity in declining populations requires well-characterized markers where patterns of selection have been documented. In vertebrates the well-studied major histocompatability complex (MHC) fulfils such a role and research on MHC loci in a population genetic and/or conservation context has burgeoned over the last decade e.g. [4], [5], [6], [7], and in plants frequency dependent selection at self-incompatibility loci is known to be capable of maintaining variation [8], [9]. However, no such standard markers have as yet been identified for invertebrates. Recently, phosphoglucose isomerase (*Pgi*) was proposed as a potentially widely applicable adaptive marker for conservation genetics in the Arthopoda [10], the most speciose animal phylum [11]. The phosphoglucose isomerase enzyme catalyzes the second step in glycolysis and shows polymorphism associated with fitness correlates in several Lepidopteran, Coleopteran and Orthopteran taxa as well as in Crustacea [reviewed in 10]. Frequently, the basis of selection on *Pgi* is linked to variation in temperature and thermal microclimate and polymorphism can be maintained either by heterozygote advantage or divergent directional selection across a cline [12], [13], [14], [15], [16], [17]. In some species clinal selection leads to one allele predominating others at particular locations along the cline [14].

It is important to acknowledge that *Pgi* is not suggested to be directly equivalent to MHC and SI loci, that there are no *a priori* expectations of *Pgi* variation, and that predicting the ratio of polymorphic to non-polymorphic species is difficult due to publication bias [10]. In this context we here investigate patterns of variation at, and selection on, *Pgi* in bumblebees (*Bombus* sp.). Bumblebees are ecologically and economically important due to their role as pollinators [18], [19], [20], yet many species are declining in range and abundance, while others remain widespread [21] and some are expanding their range e.g. [22]. Partly as a consequence, population structure and demographic events have been widely assessed in this group using neutral markers [23], [24], [25], [26], [27], [28], [29]. Together these factors make them obvious focal organisms for assessing adaptive genetic variation from both applied and theoretical conservation genetic perspectives.

## Results

**Figure 1:**
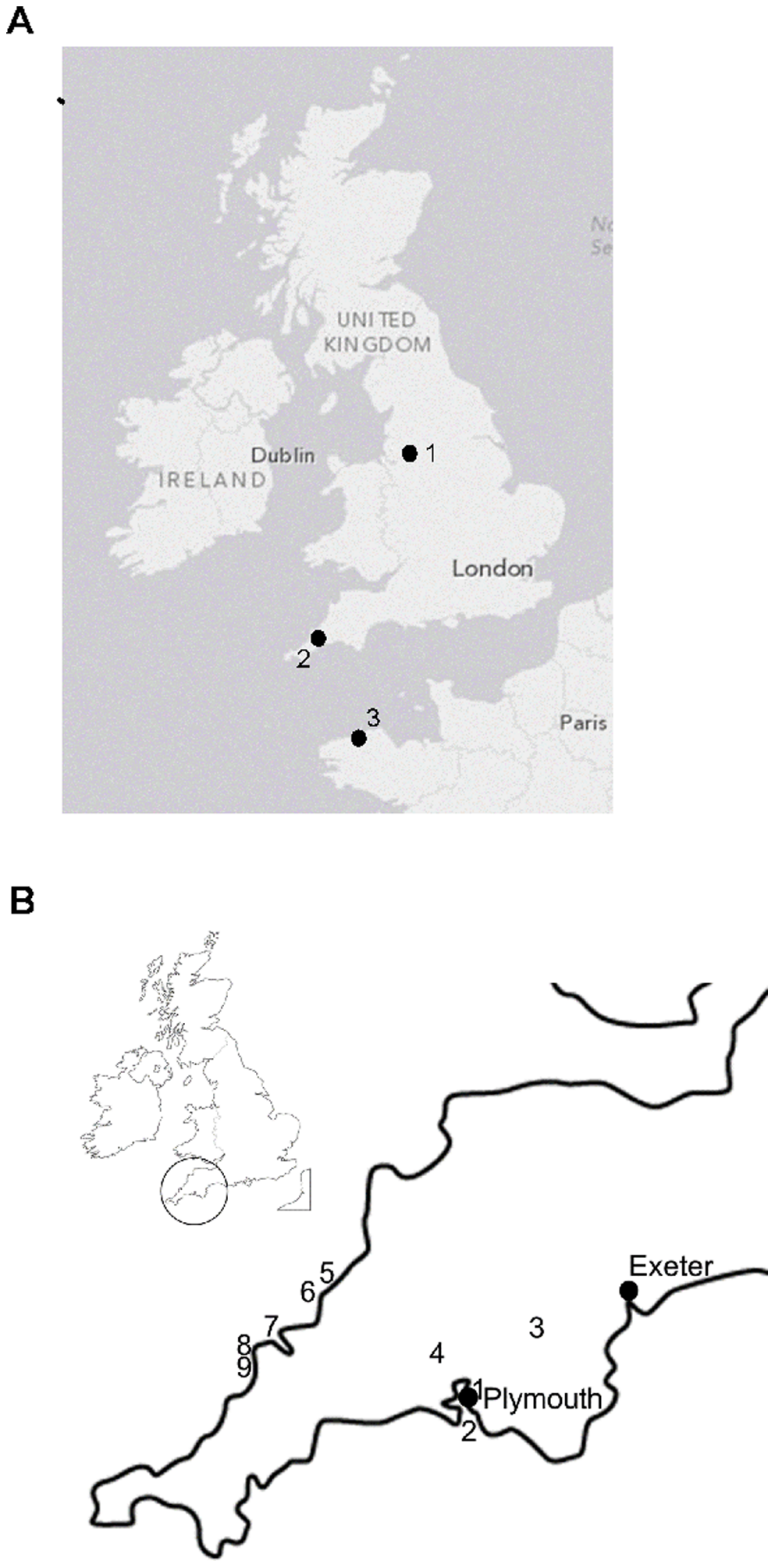
Map showing general sampling localities. (**a**) (1) Manchester, (2) South-West England, (3) Brittany; (**b**) Detailed map of sampling localities in South-West England: 1. Plymouth; 2. Wembury; 3. Dartmoor; 4. Kit Hill; 5. Boscastle; 6. Tintagel; 7. Pentire Point; 8. Trevose Head; 9. Park Head.

**Figure 2:**
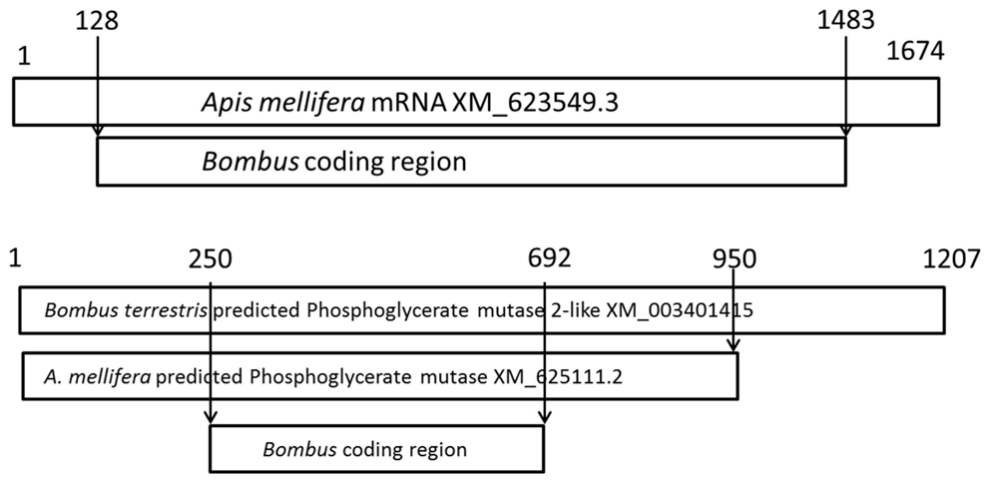
Schematic diagram representing the proportion of *Apis mellifera Pgi* mRNA covered by *Bombus Pgi* coding region sequenced in this study (above) and proportion of predicted *A. mellifera* and *B. terrestris* phosphoglycerate mutase covered by *Bombus Pgm* coding regions sequenced in this study (below). Figures indicate the base positions in the *Apis* mRNA sequence where the *Bombus* sequence begins and ends.

*Pgi* variation was assessed across five species of bumblebees sampled across south-west England (Table 1, Figure 1), representing a range of demographic histories from abundant and widespread (*Bombus lapidarius*; *B. pratorum*; *B.pascuorum*) to declining and highly localised (*B. monticola*; *B. humilis*). *B. pascuorum* and *B.* *lapidarius* were also sampled from elsewhere in the UK as well as from continental Europe (*B. pascuorum* UK, 15 samples, France 9 samples; *B. lapidarius* UK 14 samples, France 10 samples). In total 64 individual (haploid) males were sampled. Total sequence lengths varied from 2365–2396 bp including 1356 bp of coding sequence in seven exons (partial coding region obtained only, Figure 2). For comparison we also sequenced another metabolic marker, phosphoglycerate mutase (*Pgm*) for which 962–978 bp of sequence were obtained containing 441 bp of coding sequence in two exons (Genbank accessions for both loci: JQ736528–574; JQ736618–645; KC311670–KC311706). For both loci no stop codons were identified in the inferred coding regions. Similarity of the *Bombus* sequences generated with existing *Pgi* and *Pgm* sequences was confirmed by BLAST searches using the NCBI database. *Pgi* sequences (coding region only) were highly similar to existing Hymenopteran mRNA sequences (predicted *B. impatiens Pgi* coverage 100%, e-value 0, max. identity 99%; *Apis mellifera Pgi* coverage 100%, e-value 0, max. identity 99%; *Nasonia vitripennis Pgi* coverage 98%, e-value 0, max. identity 75%). *Pgm* sequences (coding region only) were also highly similar to existing Hymenopteran sequences (predicted *B. terrestris* phosphoglycerate mutase coverage 100%, e-value 0, max. identity 98%; *Apis cerana Pgm* coverage 100%, e-value 2*10^−145^, max. identity 86%; *Cotesia congregata* (Braconidae) *Pgm* coverage 99%, e-value 2*10^−84^, max. identity 76%).

**Table 1:** Locations of individuals sampled, by species.

Samples	Sampling area	Grid reference/Latitude & longitude	Date sampled	*Pgi*	*Pgm*
*B. pratorum*					
29PT.KH	Kit Hill and area, Callington	SX3771	27/05/2010		*
44PT.KH	Kit Hill and area, Callington	SX3574	01/06/2010	*	*
53PT.KH	Kit Hill and area, Callington	SX4171	01/06/2010	*	*
56PT.KH	Kit Hill and area, Callington	SX4070	01/06/2010	*	*
63PT.BT	Dartmoor	SX6881	02/06/2010	*	*
64PT.BT	Dartmoor	SX6881	02/06/2010		*
75PT.BT	Dartmoor	SX6881	02/06/2010	*	*
88PT.PL	Plymouth	SX4755	11/06/2010	*	*
01.12PT.M	Manchester	SJ8490	03/08/2012	*	
*B. monticola*					
61M.HT	Dartmoor	SX7577	23/07/2010	*	*
64M.HT	Dartmoor	SX7577	23/07/2010	*	*
65M.HMT	Dartmoor	SX7081	23/07/2010	*	*
66M.HMT	Dartmoor	SX6981	23/07/2010	*	*
67M.BT	Dartmoor	SX6881	23/07/2010	*	*
68M.BT	Dartmoor	SX6881	23/07/2010	*	*
79M.HMT	Dartmoor	SX6981	26/07/2010	*	*
81M.HMT	Dartmoor	SX6981	26/07/2010	*	*
*B. lapidarius*					
51L.HMT	Dartmoor	SX6981	19/07/2010	*	*
52L.BOS	Boscastle	SX0991	21/07/2010	*	*
54L.TTG	Tintagel	SX0488	21/07/2010	*	*
55L.PL	Plymouth	SX468551	22/07/2010	*	*
56L.BHR	Dartmoor	SX7377	23/07/2010	*	*
57L.BT	Dartmoor	SX6881	23/07/2010	*	*
58L.PL	Plymouth	SX4756	24/07/2010	*	*
1L.MAN	Manchester	SJ8490	03/08/2012	*	
2L.MAN	Manchester	SJ8490	03/08/2012	*	
3L.MAN	Manchester	SJ8490	03/08/2012	*	
4L.MAN	Manchester	SJ8490	03/08/2012	*	
5L.MAN	Manchester	SJ8490	03/08/2012	*	
6L.MAN	Manchester	SJ8490	03/08/2012	*	
8L.MAN	Manchester	SJ8490	03/08/2012	*	
1L.FR	Brittany, France	^(a)^Samples collected between Primél-Trégastel (48.716564, −3.81922) and Locquirec (48.687561, −3.671494)	25/08/2012	*	
2L.FR	Brittany, France	Ditto	25/08/2012	*	
3L.FR	Brittany, France	Ditto	25/08/2012	*	
4L.FR	Brittany, France	Ditto	25/08/2012	*	
5L.FR	Brittany, France	Ditto	25/08/2012	*	
6L.FR	Brittany, France	Ditto	25/08/2012	*	
7L.FR	Brittany, France	Ditto	25/08/2012	*	
8L.FR	Brittany, France	Ditto	25/08/2012	*	
9L.FR	Brittany, France	Ditto	25/08/2012	*	
10L.FR	Brittany, France	Ditto	25/08/2012	*	
*B. pascuorum*					
50PC.PL	Plymouth	SX4554	28/07/2010	*	*
51PC.PL	Plymouth	SX4554	28/07/2010	*	*
52PC.PL	Plymouth	SX4555	28/07/2010		*
60PC.PL	Plymouth	SX4755	28/07/2010		*
61PC.PL	Plymouth	SX4755	28/07/2010	*	*
63PC.PL	Plymouth	SX4756	28/07/2010	*	*
66PC.PL	Plymouth	SX4755	28/07/2010	*	*
68PC.TH	Trevose Head	SW8675	30/07/2010		*
1PC.MAN	Manchester	SJ8490	03/08/2012	*	
2PC.MAN	Manchester	SJ8490	03/08/2012	*	
3PC.MAN	Manchester	SJ8490	03/08/2012	*	
4PC.MAN	Manchester	SJ8490	03/08/2012	*	
5PC.MAN	Manchester	SJ8490	03/08/2012	*	
6PC.MAN	Manchester	SJ8490	03/08/2012	*	
7PC.MAN	Manchester	SJ8490	03/08/2012	*	
8PC.MAN	Manchester	SJ8490	03/08/2012	*	
9PC.MAN	Manchester	SJ8490	03/08/2012	*	
10PC.MAN	Manchester	SJ8490	03/08/2012	*	
1PC.FR	Brittany, France	^(a)^Samples collected between Primél-Trégastel (48.716564, −3.81922) and Locquirec (48.687561, −3.671494)	25/08/2012	*	
2PC.FR	Brittany, France	Ditto	25/08/2012	*	
3PC.FR	Brittany, France	Ditto	25/08/2012	*	
4PC.FR	Brittany, France	Ditto	25/08/2012	*	
5PC.FR	Brittany, France	Ditto	25/08/2012	*	
6PC.FR	Brittany, France	Ditto	25/08/2012	*	
7PC.FR	Brittany, France	Ditto	25/08/2012	*	
8PC.FR	Brittany, France	Ditto	25/08/2012	*	
9PC.FR	Brittany, France	Ditto	25/08/2012	*	
*B. humilis*					
43HU.TTG	Tintagel	SX0488	21/07/2010		*
47HU.TH	Trevose Head	SW8675	30/07/2010	*	*
48HU.TH	Trevose Head	SW8675	30/07/2010		*
50HU.TH	Trevose Head	SW8675	30/07/2010		*
51HU.TH	Trevose Head	SW8675	30/07/2010		*
61HU.TH	Trevose Head	SW8470	30/07/2010		*
66HU.PH	Park Head	SW8470	30/07/2010		*
69HU.PH	Park Head	SW8471	30/07/2010	*	*
*B. hortorum*					
45HR.TTG	Tintagel	SW0589	09/07/2010		*
48HR.PL	Plymouth	SX4756	24/07/2010		*
49HR.PL	Plymouth	SX4555	28/07/2010		*
51HR.PL	Plymouth	SX5054	28/07/2010		*
53HR.PL	Wembury	SX4948	28/07/2010		*
54HR.WP	Wembury	SX4948	28/07/2010		*
59HR.PL	Plymouth	SX4755	29/07/2010		*
60HR.PH	Park Head	SW8470	30/07/2010		*

Grid reference is the Ordnance survey in 4-figure format (note that for localities with the same grid reference this refers to a specific 1 km square, not a single point). Dates are in standard UK format (day/month/year). See note in the text regarding likely sampling of individuals from the same nest. An asterisk in the columns headed *Pgi* and *Pgm* indicates which samples were sequenced. ^(a)^ Exact sample locations for each individual were not recorded. However, individuals were sampled at multiple sites between the two locations indicated (all separated by >200 m) and not more than a few individuals were collected from a single locality.

**Figure 3:**
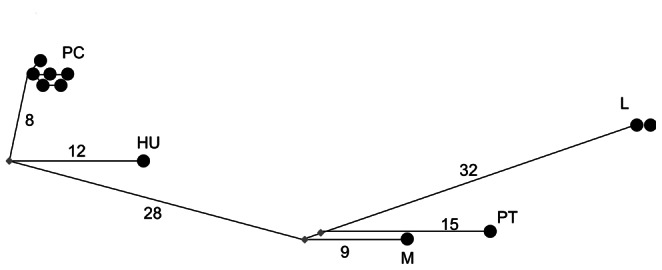
Network showing the relation between phosphoglucose isomerase haplotypes for all regions (coding and non-coding). PC = *B. pascuorum*, HU = *B. humilis*, PT = *B. pratorum*, M = *B. monticola*, L = *B. lapidarius.* Numbers along the connecting lines indicate the number of mutated positions between haplotypes. Nodes are not proportional to haplotype frequencies.

**Figure 4:**
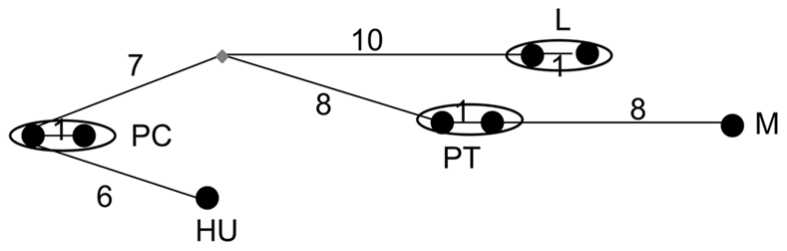
Network showing the relation between phosphoglucose isomerase haplotypes for coding regions only. PC = *B. pascuorum*, HU = *B. humilis*, PT = *B. pratorum*, M = *B. monticola*, L = *B. lapidarius.* Numbers along the connecting lines indicate the number of mutated positions between haplotypes. Nodes are not proportional to haplotype frequencies.

**Figure 5:**
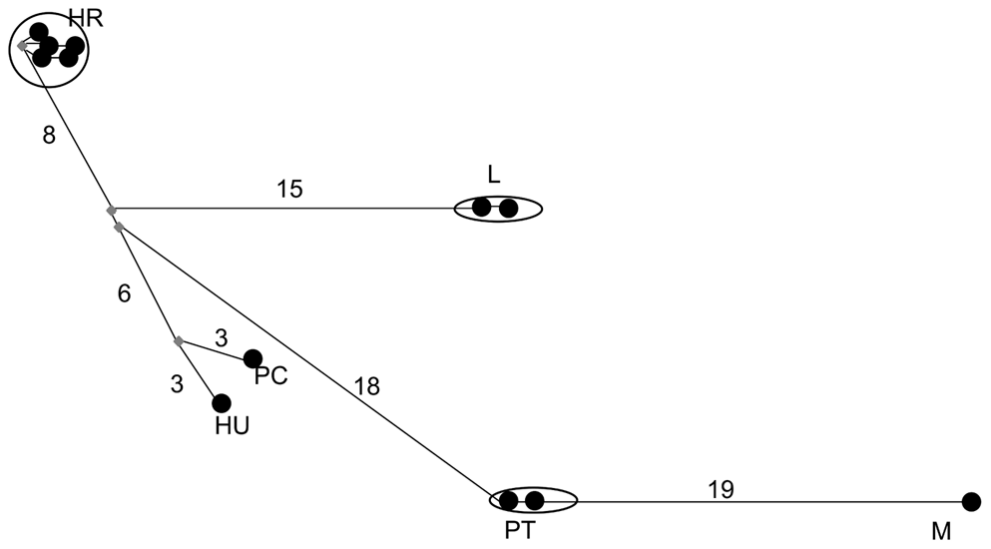
Network showing the relation between phosphoglycerate mutase haplotypes for all regions (coding and non-coding). HR = *B. hortorum*, PC = *B. pascuorum*, HU = *B. humilis*, PT = *B. pratorum*, M = *B. monticola*, L = *B. lapidarius.* Numbers along the connecting lines indicate the number of mutated positions between haplotypes. Nodes are not proportional to haplotype frequencies.

**Figure 6:**
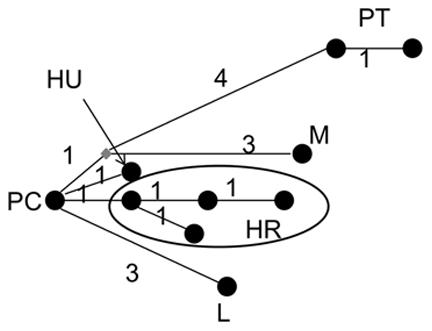
Network showing the relation between phosphoglycerate mutase haplotypes for coding regions only. HR = *B. hortorum*, PC = *B. pascuorum*, HU = *B. humilis*, PT = *B. pratorum*, M = *B. monticola*, L = *B. lapidarius.* Numbers along the connecting lines indicate the number of mutated positions between haplotypes. Nodes are not proportional to haplotype frequencies.

Very few non-coding segregating sites were observed for either locus in any species (Table 2), although a microsatellite sequence was observed in *Pgi* in *B. pratorum.* In coding regions, a maximum of one segregating site (synonymous or non-synonymous) was observed in *B. pascuorum*, *B. pratorum* and *B. lapidarius* for *Pgi* and likewise few were observed for *Pgm* (Table 2). For comparison, estimates from *Drosophila*, where diversity is also low [10], are also provided (Table 2). Nucleotide diversity is similar for *D. melanogaster* and *D. yakuba,* but greater for *D. simulans*. The lack of intra-specific variation observed precluded assessment of selection by McDonald-Kreitman (MK) tests in most cases [30]; however, the number of fixed inter-specific non-synonymous and synonymous differences were calculated for completion. (Note that synonymous sequence divergence between species pairs varied from 1.7% (*B. pascuorum* & *B. humilis*) to 5.1% (*B. lapidarius* & *B. pratorum*) at *Pgi* and from1.0% (*Bombus pascuorum* & *B. humilis*) to 7.9% (*B. lapidarius* & *B. pratorum*) at *Pgm*. For *Drosophila melanogaster* & *D. simulans* synonymous site divergence at *Pgi* was 7.3%)). No large excess of non-synonymous substitutions was observed for any comparison at any locus and overall very little inter-specific divergence was observed, especially at non-synonymous sites (Table 3, Figures 3, 4, 5, 6). These results obviate the use of other tests of selection since within-species variation is so severely limited. In total across all species only 11 haplotypes were observed for complete sequences (including non-coding regions). When only coding regions were considered 8 haplotypes were observed. The relationship between haplotypes matched the known species phylogeny (Figures 3, 4, 5, 6). Across all species, 12 amino acid differences were observed (Table 4).

**Table 2:** Summary data for *Pgi* and *Pgm* loci across *Bombus* species and for three *Drosophila* species at *Pgi* for comparison (from unpublished sequences in GenBank – see methods).

				Segregating sites			Nucleotide diversity		
	n	Total length (bp)	Coding length	Non-coding	S_S_	S_A_	All coding	π_S_	π_A_
***Pgi***									
*B. pratorum*	6	2365	1356	0	1	0	0.0002	0.0010	0.0000
*B. monticola*	8	2396	1356	0	0	0	0.0000	0.0000	0.0000
*B. lapidarius*	24	2374–2375	1356	1	1	0	0.0001	0.0002	0.0000
*B. pascuorum*	24	2394–2396	1356	5	0	1	0.0001	0.0000	0.0001
*B. humilis*	2	2371	1356	0	0	0	0.0000	0.0000	0.0000
*D. melanogaster*	6	n/a	1665	n/a	1	2	0.0006	0.0009	0.0005
*D. simulans*	11	n/a	1665	n/a	7	2	0.0015	0.0057	0.0003
*D. yakuba*	13	n/a	1665	n/a	1	0	0.0001	0.0004	0.0000
***Pgm***									
*B. pratorum*	8	968	441	0	1	0	0.0012	0.0055	0.0000
*B. monticola*	8	967	441	0	0	0	0.0000	0.0000	0.0000
*B. lapidarius*	7	978	441	1	0	0	0.0000	0.0000	0.0000
*B. pascuorum*	8	977	441	0	0	0	0.0000	0.0000	0.0000
*B. humilis*	8	977	441	0	0	0	0.0000	0.0000	0.0000
*B. hortorum*	8	962–972	441	2	2	1	0.0023	0.0052	0.0016

S_S_, synonymous sites; S_A_ non-synonymous sites. π_S_, π_A_ nucleotide diversity per synonymous and non-synonymous site respectively. *B. hortorum* could not be sequenced at *Pgi* due to poor quality PCR amplification. Coding regions were identified as outlined in the text. N.B. n = number of haploid males sampled.

**Table 3:** Number of fixed synonymous and non-synonymous interspecific differences between representative species pair comparisons for *Pgi* (1356 bp) and *Pgm* (441 bp).

	Intraspecific differences		Fixed interspecific differences		G-test	
	Synonymous	Non-synonymous	Synonymous	Non-synonymous	G	*P*
***Pgi***						
*B.pascuroum & B.humilis*	0	1	5	1	n.a	n.a
*B.pratorum & B.monticola*	1	0	7	1	n.a	n.a
*B.pascuroum & B.lapidarius*	1	1	9	8	0.006	0.94
*B.pascuroum & B.pratorum*	1	1	7	8	0.008	0.93
*B.lapidarius & B.pratorum*	2	0	14	4	n.a	n.a
*D. melanogaster & D. simulans*	8	4	25	0	n.a	n.a
***Pgm***						
*B.pascuroum & B.humilis*	0	0	1	0	n.a	n.a
*B.pratorum & B.monticola*	1	0	4	2	n.a	n.a
*B.pascuroum & B.lapidarius*	2	1	3	0	n.a	n.a
*B.pascuroum & B.pratorum*	1	0	4	1	n.a	n.a
*B.lapidarius & B.pratorum*	1	0	7	1	n.a	n.a
*B.lapidarius & B.hortorum*	0	0	4	0	n.a	n.a

Note the difference in sequence length between *Pgi* and *Pgm* (see π in Table 1). Estimates for *D. melanogaster* and *D. simulans* are provided for comparison (synonymous site divergence for this pair is similar to the maximum distance between *Bombus* pairs – see text). Comparisons were made between pairs of subgeners (*B. pascuorum* & *B. humilis; B. pratorum* & *B. monticola*) as well as an arbitrary selection of common species pairs to illustrate the full range of differences observed. The lack of intraspecific differences observed precludes McDonald-Kreitman (30) tests of selection in most cases.

**Table 4:** Location of amino acid replacements (relative to amino acid sequence alignment position – note *Pgi* sequence is partial) across species for phosphoglucose isomerase (amino acid sequence based on inferred coding regions, hence putative replacement).

Amino acid position	Putative replacement	Species or individuals with replacement
9	glutamic acid to aspartic acid	*B. pascuorum* & *B. humilis*
32	glutamic acid to glutamine	*B. pascuorum* & *B. humilis*
50	glutamic acid to lysine	*B. pratorum* & *B. monticola*
77	serine to asparagine	*B. pascuorum* & *B. humilis*
140	arginine to glutamine	*B. lapidarius*
184	serine to cysteine	*B. pascuorum* – one individual only (sample 1FR)
196	glutamic acid to lysine	*B. humilis*
262	tyrosine to histidine	*B. pascuorum* & *B. humilis*
278	valine to isoleucine	*B. pascuorum* & *B. humilis*
285	valine to isoleucine	*B. pascuorum* & *B. humilis*
407	alanine to threonine	*B. lapidarius*
416	proline to leucine	*B. pratorum*

Unless otherwise stated, replacements were observed for all samples within each species named.

## Discussion

Wheat [10] has reviewed the characteristics of a general adaptive marker for invertebrate conservation genetics, with special reference to *Pgi*. As outlined [10], a general adaptive marker in this context must show genetic variation that affects fitness, there should be a functional understanding of this across a range of taxa, and necessarily an adaptive marker must be heterozygous within populations. Here a lack of variation in *Pgi* was observed within bumblebee species with the low observed number of segregating sites precluding robust tests of selection. Thus, we suggest that *Pgi* is not a useful marker of functional variation in a conservation context in these taxa. Below we first consider whether or not selection can be expected to maintain variation in haplodiploid insects and thus whether or not it is realistic to expect variation at functional loci in these taxa, before exploring other caveats and hypotheses of relevance to this study.

At functional loci, balancing selection can maintain variation by various different processes including heterozygote advantage (overdominance), frequency-dependent selection and variations in the direction and pattern of selection in space [31]. There is no *a priori* reason to expect frequency-dependence to be less effective in haplodiploid than diploid systems [32]. However, the modes of selection found to affect *Pgi* polymorphism are either heterozygote advantage (for example in butterflies [10], [33], [34], [35]) or directional selection along a cline [14], [15].

The conditions under which heterozygote advantage can bring about balanced polymorphisms in haplodiploids are more restricitive than those within diploid systems [32], [35]. This can be realised by considering that directional selection in the haploid sex can overcome heterozygote advantage in the diploid sex [36]. However, whilst more restrictive than for diploids, there are still conditions for which a balanced polymorphism can be maintained by heterozygote advantage in haplodiploids, depending on the strength of directional selection on haploid genotypes [36]. Polymorphism can also be maintained in haplodiploids when selection affects male and female genotypes differently [36]. Speculatively this is perhaps unlikely for a gene such as *Pgi* (catalysing the second-step in glycolysis) in bumblebees. Thus considering a hypothetical single *Pgi* locus with two alleles in a haplodiploid system, whether or not heterozygote advantage in females would maintain variation would depend on each particular allele not being selected against too strongly in males [36]. Data obtained here cannot be used to evaluate the likelihood of this scenario.

Directional selection across a cline has also been reported to maintain variation at *Pgi* in some species (linked to climatic variation across the cline [10], [14], [15]). Detecting multiple alleles at a locus in this case would depend on sampling the part of a species range where multiple alleles would be expected to occur, for example at the ‘climatic centre’ of the range. For all of the species studied here, the UK and northern France is approximately in the middle of their range between northern and southern limits (data available from Natural History Museum, London, http://www.nhm.ac.uk/research-curation/research/projects/bombus/index.html). If this range mid-point coincides with a mid-point of climatic range then this is where multiple allelic forms would be expected to occur [14]. Metzger *et al.* [37] have produced a climatic stratification of Europe. With regard to temperature gradient, samples from South-West England and Brittany are in the mid-point on this gradient, with Manchester also being close to the mid-point of the gradient, but at a slightly cooler grade than the former locations [37]. Consequently, it seems reasonable to assume that samples were obtained from the part of the range where multiple allelic forms would be expected to occur if divergent directional selection at different parts of the range affected variation for *Pgi* in bumblebees. However, to establish this it would be necessary to sample comprehensively across the entire range to examine whether or not *Pgi* variation is associated with a gradient of temperature across Europe (which was not possible within the constraints of this study).

The existence of balanced polymorphism in Hymenoptera has been demonstrated for the *Pgm-3* locus in ants (*Solenopsis invicta* [38]). In that case this is maintained by a balance between gene-flow and directional selection in colonies with different life-history strategies (monogyne versus polygyne colonies [38]). While this specific scenario is not relevant to bumblebees it demonstrates that there are mechanisms by which balanced polymorphisms can be maintained in haplodiploid taxa.

The degree of genetic variation observed within haplodiploid insects and in social Hymenoptera, in particular, is expected to be comparatively low due to evolutionary genetic consequences of haplodiploidy and other factors associated with sociality [32], [36], [39]. Reviews find that haplodiploids, and eusocial Hymenoptera especially, clearly have lower diversity than other insects at allozyme loci [36], [40], although this pattern does not hold at microsatellite loci due to their higher mutation rate [36]. Bumblebees might be further expected to have low genetic diversity since most species examined are monoandrous [40], [41]. Surveys of mitochondrial DNA in *B. terrestris* and *B. pascuorum* in Europe reveal low levels of genetic diversity [23], [24], [25] as do surveys of internal transcribed spacer-2 of *B. ardens* in Korea [42]. Thus the general lack of variation observed at *Pgi* is perhaps unsurprising in this context.

Other hypotheses and caveats regarding the lack of variation at *Pgi* in bumblebees observed here relate to sample size, demographic effects, linkage, selective sweeps and divergence time and the ability of bumblebees to thermoregulate (discussed below).

In an evolutionary genetics context there are two reasons why the sample size here is more robust than it may initially appear [43]: (i) evolutionary genetic approaches compare samples to estimate the relative properties of nucleotides as opposed to the entire sequence (e.g. examining the probability that nucleotides are polymorphic), in which case the number of observations is related to the length of the sequence; (ii) alleles sampled are not statistically independent of one another i.e. because alleles are related to one another, there is a diminishing chance of finding more segregating sites as more alleles are sequenced [43]. According to Gillespie [43] the mean number of segregating sites is proportional to the logarithm of the sample size. Thus if we had tripled our sample size to 72 rather than 24 sequences the expected number of segregating sites would only have increased by a factor of 1.34 [43].To reiterate: across all samples there was both a low level of inter-specific divergence at non-synonymous sites as well as a lack of intra-specific variation. These values are similar to those obtained for unpublished sequences for *Drosophila* species downloaded from GenBank, where there is also a lack of variation (Table 2). Since males were sampled (whose flight range significantly exceeds workers, at least in *B. terrestris* [44]) it is unlikely that multiple individuals from single nests were sampled. (When sampling workers, multiple individuals from single nests are infrequently sampled when sites are separated by ∼200 m and only a very few individuals are taken from a single locality, as sampled here [see e.g. 45]). Note also that a maximum of two coding haplotypes were observed across all samples from Brittany to northern England and from two separate generations sampled (2010 and 2012).

Since we do not have data for the entire *Pgi* region in *Bombus* we cannot eliminate the possibility that specific codons outside of the region sequenced may be under balancing or directional selection. In comparison with the honey bee, the 1356 bp coding region that we have sequenced in *Bombus* represents ∼80% of the predicted *Apis Pgi* sequence (Figure 2), thus it is still possible that *Bombus Pgi* is variable, but we have not detected that variation (our sequences were shorter due to primer design constraints – see materials and methods). However, by cross-referencing to *Melitaea cinxia* mRNA sequences it was possible to obtain a coding region for *Bombus* that corresponded to a region containing nine of the thirteen segregating amino acid sites known in the Finnish metapopulation of this butterfly [46] including two SNP sites that exhibit strong and significant departures from Hardy-Weinberg equilibrium due to heterozygote excess in this species. Thus we consider it a highly unlikely scenario that variation leading to a signal of balancing selection would occur exclusively outside of the gene region sequenced.

Regarding demographic effects, the species sampled here include a range of recent demographic histories in the UK (from rare and declining [*B. monticola* and *B. humilis*] to currently ubiquitous [all other species]). Panmixis is likely for the currently ubiquitous species since estimates of genetic differentiation based on neutral loci are very low [e.g. 27]. Across both rare and ubiquitous species, a similar lack of *Pgi* and *Pgm* variation is found. For two of the common focal species, *B. pascuorum* and *B. lapidarius,* samples were obtained from continental Europe as well as the UK, and the degree of variation remained low with no notable patterns regarding the distribution of genetic variation between different sampling localities. Thus it seems unlikely that the lack of variation observed in UK samples is due to a founder effect resulting from colonisation of the UK. It is still possible, however, that some other demographic effect may account for the lack of variation at *Pgi* in *Bombus*. For example, the lack of variation in *B. pascuroum* described above [25] is consistent with colonization of northern Europe from a single glacial refugium. Regardless, this does not negate the main message of this study which is that *Pgi* is not useful as a conservation genetic marker in these taxa.

Since *Pgi* polymorphism is linked to a trade-off between thermal stability of some allelic forms of the enzyme versus kinetic advantage of others [14], [35] expectations regarding the nature of selection and polymorphism at *Pgi* should perhaps take into account the ability of invertebrates to thermoregulate. Bumblebees can thermoregulate physiologically, either by shivering [47] and possibly by a ‘futile cycle’ of substrate cycling [48] although this is contentious [49]. Thoracic temperature can also be maintained by the petiole between the thorax and abdomen acting as a counter-current heat exchanger [50]. In contrast, butterfly thermoregulation occurs by behavioural means (various forms of basking, [51]) and thus is dependent on external environmental conditions. In the beetle, *Chrysomella aenicollis* where *Pgi* variation has also been noted [14], [15], body temperature has been shown to closely follow air temperature [14]. Allozyme variation for *PGI* has also been documented in crickets [52]. As in butterflies, Orthopterans display behavioural thermoregulation [53], [54]. It is interesting to note that Riddoch [13] observed that *PGI* allozyme variation was often noted in stressful situations where there was one or a combination of increased temperature, salinity, desiccation risk or reduced oxygen. Speculatively, the physiological thermogenesis and thermoregulation of groups such as bumblebees and moths may remove the selective pressure for variation of *Pgi* to be maintained, since individuals showing the most kinetically advantageous form of the enzyme will be favoured by selection as the vagaries of external climate on flight capability are mitigated by the other adaptations that have evolved. This is countered, however, by the observation that *Pgi* variability has been documented in mammals e.g. [55] which have very well developed physiological thermoregulation and by the similar values of variation obtained for *Drosophila* (see above).

An alternative explanation for the observed lack of variation is that the *Pgi* locus in bumblebees is linked to a region of the genome that is under (non-balancing) selection, in which case polymorphism would be expected to be minimal or reduced by the action of a selective sweep see [56] for a review. Given the observed lack of variation in multiple species this is unlikely unless the *Pgi* gene is located in a large non-recombining region of the bumblebee genome. We currently have no data on the location of the *Pgi* gene in *Bombus* that we can use to investigate the validity of this. Low divergence at synonymous sites could also be a consequence of low divergence time or low mutation rate. However divergence times are not recent: interpreting published figures [57] gives approximate divergence times of 13Mya for *B. pratorum* and *B. monticola* (subgenus *Pyrobombus*), 5–6Mya for *B. pascuorum* and *B. humilis* (subgenus Thoracobombus), 22Mya for the lineage containing *B. lapidarius* (subgenus *Melanobombus*) from the lineage containing *Pyrobombus* and 23–24Mya for the lineage containing *Thoracobombus* from the lineage containing *Pyrobombus* and *Melanobombus*.

In summary, *Pgi* is polymorphic in many species, it is associated with fitness in numerous species and there is a functional understanding of *Pgi* variation in these cases – thus in many instances *Pgi* clearly has a role as a useful adaptive marker [10]. However, in bumblebees this is not the case. The most parsimonious explanations for this lack of variation at *Pgi* in these taxa are the restrictive conditions for overdominance, or historical demographic effects explain the general lack of variation at *Pgi* in bumblebees. Whether other loci would provide alternatives for a general adaptive marker in the Arthropoda is an open question.

## Materials and Methods

### Study Species

Samples of four ubiquitously distributed species (*Bombus lapidarius* (Linnaeus), *Bombus hortorum* (Linnaeus), *Bombus pascuorum* (Scopoli) and *Bombus pratorum* (Linnaeus)) and two declining species (*Bombus humilis* (Illiger) & *Bombus monticola* Smith; [58], [59]) were collected in south-west England in the spring and summer of 2010 (Table 1). Additional samples of *B. pascuorum* and *B. lapidarius* and one sample of *B. pratorum* were collected from the city of Manchester, north-west England in summer 2012 and from Brittany, France, between Primél-Trégastel and Locquirec, also in 2012 (Table 1, Figure 1). In the UK, *B. pascuorum* and *B. humilis* represent a common and declining species pair within the same subgenus (*Thoracobombus*) respectively, as do *B. pratorum* and *B.* *monticola* (*Pyrobombus*). Permission for sampling declining species was obtained from Natural England as well as the managers of each sampling locality. Samples of *B. humilis* were taken from a stretch of the north coast of Cornwall from Park Head (Ordnance survey grid reference [OSGR] SW8470; latitude 50.49742, longitude –5.04602) to Boscastle (OSGR: SX0991; latitude 50.68668, longitude - 4.70425). *B. humilis* is sparsely linearly distributed across this area being confined to suitable coastal habitats in this region [60]. Samples of *B. monticola* were taken from a single 10-by-10 km square area within Dartmoor National Park (table 1) and are thus likely to represent a single panmictic population. All other species are ubiquitous across the UK, thus samples taken from within south-west and north-west England were assumed to originate from a single panmictic population (see previous estimates of *F*_ST_ in *B. pascuorum*, [27]). Males were used for genetic analyses (see below). Males are useful for the genetic analyses used here since they are haploid and thus eliminate the need for haplotype phase inference that is required from diploid data. Some of these samples were also used for another study of innate immune system variation [61].

### DNA Extraction, PCR Development, and Sequencing

DNA was extracted using a modified ammonium acetate protocol [62] (protocol available on request).

For phosphoglucose isomerase (*Pgi*) overlapping primer sets were developed to amplify and sequence a 5′ and 3′ region separately. For the 3′ region, initial primers were developed from an alignment of *Apis mellifera Pgi* mRNA sequence (accession number XM623549.2) with Lepidopteran sequences (*Euphydryas aurinia* GU2134322.1; *Melitaea cinxia* EU888473.1). These primers (details available on request from the authors) allowed sequence to be obtained from *Bombus pratorum*. This sequence was then used to generate *Bombus*-specific primers for the 3′ region (forward GTCCTTTAATGGTAACTGAAGC; reverse AATTGATATCCCAAATAATCCCTTG). PCR conditions were: 94°C for 3 minutes (mins) followed by 35 cycles of 94°C for 30 s, 50°C for 45 s, 72°C for 3 mins 30 s and by a final extension of 72°C for 10 mins. Products were amplified in a 20 µl reaction volume containing 1 unit of *Taq* polymerase, 2 µl 10X reaction buffer, 0.5 µM each primer, 0.25 mM each dNTP, 2 µl template DNA (extracts unquantified), 2 mM MgCl_2_ and 12.9 µl water using a Qiagen core kit (West Sussex, UK).

For the 5′ region initial primers were identified from an alignment of *Apis mellifera* mRNA (XM623549.2) with *Bombus impatiens* mRNA (JI121890.1). Initial sequences obtained from PCR using these primers allowed the development of a second forward primer, internal to the first primer. PCR products were then generated in a semi-nested PCR design using these primers. Conditions for the first-step were 94°C for 3 mins followed by 30 cycles of 94°C for 30 s, 52°C for 30 s, 72°C for 2 mins followed by a final extension of 72°C for 10 mins. Products were amplified in a 20 µl reaction volume containing 1 unit of *Taq* polymerase, 2 µl 10X reaction buffer, 0.2 µM each primer, 0.2 mM each dNTP, 2 µl template DNA (extracts unquantified), 2 mM MgCl_2_ and 14.2 µl water using a Qiagen core kit (West Sussex, UK). Primer sequences were: forward CCGAAGCGGCATGGACTAA (Pgi 5′ F2), reverse CCAAAAGCCATATTTTAGCAGAAG (Pgi 5′ R3B). The second PCR used the same reverse primer, but the sequence of the forward primer was CAACAAAATCCTAAACGCTTCG (Pgi 5′ F3). Cycle conditions were the same as for the first PCR, except 35 cycles were performed. Reaction mixes were also the same except 1 µl of PCR product from the first reaction was used as template and the volume of water was adjusted accordingly.

For additional samples from Manchester and Brittany, the PCR protocol for the 5′ *Pgi* region was slightly altered as follows. Products were amplified in a 20 µl reaction volume containing 1 unit of *Taq* polymerase, 2 µl 10X reaction buffer, 0.5 µM each primer, 0.2 mM each dNTP, 1 µl template DNA (extracts unquantified), 2 mM MgCl_2_ and 14 µl water using a Qiagen core kit (West Sussex, UK). Cycle conditions remained the same. The second PCR was then fully nested using Pgi 5′ F3 (sequence above) and an internal reverse primer Pgi 5′ R2 (CCATCTATGTTACTAACAAAATGAAC). Products were amplified in a 20 µl reaction volume containing 1 unit of *Taq* polymerase, 2 µl 10X reaction buffer, 0.2 µM each primer, 0.2 mM each dNTP, 0.5, 1 or 2 µl DNA (from the first round of PCR), 2 mM MgCl_2_ with the amount of water adjusted accordingly, again using a Qiagen core kit (West Sussex, UK). Cycle conditions remained the same as above. Development of this fully nested PCR required the development of a final primer pair and PCR to ‘bridge’ a resultant gap between the 5′ Pgi and 3′ Pgi fragments amplified separately as described above (full details available on request). These primers were forward: TAATATTGGAATTGGTGGTTCAG and reverse: AAAAAGAGTTGTTTCTGGATTCAA. Cycle conditions were 94°C for 3 mins followed by 35 cycles of 94°C for 30 s, 52°C for 45 s, 72°C for 3 mins followed by a final extension of 72°C for 10 mins. Products were amplified in a 20 µl reaction volume containing 1 unit of *Taq* polymerase, 2 µl 10X reaction buffer, 0.5 µM each primer, 0.25 mM each dNTP, 2 µl template DNA (extracts unquantified), 2 mM MgCl_2_ and 12.9 µl water using a Qiagen core kit (West Sussex, UK).

For phosphoglycerate mutase (*Pgm*) primers (forward CGTCATGGAGAAAGTGAATGG, reverse CCCTTCTTTTAATTGAGGAATAATA) were developed from an alignment of *Apis mellifera* mRNA (XM625111.2) with the braconid wasp *Cotesia congregrata* (AM492673.1). PCR conditions were 94°C for 3 mins followed by 35 cycles of 94°C 30 s, 48°C for 30 s, 72°C for 2 mins followed by a final extension of 72°C for 10 mins. Products were amplified in a 20 µl reaction volume containing 1 unit of *Taq* polymerase, 2 µl 10X reaction buffer, 0.5 µM each primer, 0.2 mM each dNTP, 2 µl template DNA (extracts unquantified) and 14.2 µl water using a Qiagen core kit (West Sussex, UK). All alignments of mRNAs described above were framed against available *Apis mellifera* genomic DNA (to avoid accidental development of primers across different exons).

Sequencing of initial PCR products during development was carried out ‘in-house’ using a standard cycle sequencing reaction (96°C for 1 min, then 30 cycles of 96°C for 10 s, 50°C for 5 s, 60°C for 4 min). Sequencing reactions contained one-eighth final concentration Big-Dye terminator v3.1 ready reaction mix (Applied Biosystems, Warrington, UK), 3.5 µl sequencing buffer, 8.5 µl water and 2 µl PCR product. All other sequencing was outsourced to Macrogen Europe, Amsterdam. Prior to sequencing, PCR products were either run out by agarose gel electrophoresis and bands excised and cleaned using a Qiaquick gel extraction kit (Qiagen, Crawley, West Sussex UK) or were cleaned using an ExoI and shrimp alkaline phosphatase protocol (available on request).

All sequence alignments were made in BioEdit 7.0.5.3 [63] using Clustal W [64]. Sequences were then imported into DNAsp [65] for further analysis. For *Pgm*, coding regions were assigned by aligning genomic DNA sequences with available information for *Bombus terrestris* mRNA (XM003401415.1) and *Apis mellifera* phosphoglycerate mutase mRNA (XM625111.2). For *Pgi* we were able to infer coding regions and the correct reading frame by alignment with *B* ***.*** *impatiens* mRNA sequences (JI121890.1) and predicted *Apis mellifera Pgi* mRNA sequences (XM623549.2). Intronic sequences were then manually spliced out and coding regions translated to confirm the absence of stop codons (confirming the inferred reading frame was correct). Further analyses were made using these coding regions in DNAsp (65). Basic measures of sequence variation such as nucleotide diversity (π), total number of segregating sites and number of segregating sites in non-coding and coding regions were estimated. Other intra-specific variation was also recorded from sequence alignments such as the presence of microsatellites and insertion/deletion events (indels) in intronic regions.

Overall, a lack of intra-specific variation was observed at all loci (see Results) and few inter-specific differences were observed. Consequently, statistical estimates of selection (e.g. Ka/Ks ratios, Tajima’s D [66]) are limited by a lack of power and hence are not calculated. For completion we provide tables of the number of fixed synonymous and non-synonymous interspecific differences, calculated in DNAsp [65]. Given the lack of intraspecific segregating sites observed, McDonald-Kreitman [30] tests and calculation of their significance was not applicable in most cases. We emphasise, however, that observing a lack of variation at this locus both addresses our original aims and is indeed notable due to the variability of this locus (for the same region sequenced here) in other taxa and the proposal of it as a general marker of functional variation in the Arthropoda [10] (see Discussion). For comparison, estimates of nucleotide diversity across all sites and for synonymous and non-synonymous sites only were also calculated for *Drosophila melanogaster*, *D. simulans* and *D. yakuba,* where variation is also known to be low [10], from unpublished data in GenBank; (L27544–L27546, L27553–L27555 [*D. melanogaster*], L27547–L27551, U20557–U20559 [*D. simulans*], L27673–L27685 [*D. yakuba*] [67]). A McDonald-Kreitman test was calculated for *D.melanogaster*-*D. simulans* only (other pairwise synonymous site diversity values were all much greater than 0.1). DNAsp cannot handle non-standard codes used to identify base ambiguities (e.g. Y, R, M) so these were removed from sequences prior to analysis. Consequently, estimates of variation and divergence at *Pgi* for these species are conservative.

Haplotype networks were constructed in Network 4.6.1.0 (fluxus-engineering.com, [68]). DNAsp [65] was used to convert sequence alignments into ‘Roehl format’, deleting invariant sites. Networks were constructed with the median joining method and nodes were not plotted proportional to frequencies.
